# Identification of the bHLH Transcription Factor Family in Orah Mandarin and the Response of *CrbHLH46* to Low-Temperature Stress

**DOI:** 10.3390/plants14060882

**Published:** 2025-03-12

**Authors:** Chaoying Chen, Xulin Li, Ke Wen, Tuo Yin, Ping Tian, Ke Zhao, Li Zhang, Xianyan Zhou, Xiaozhen Liu, Hanyao Zhang

**Affiliations:** 1 Yunnan Provincial Key Laboratory for Conservation and Utilization of In-forest Resources, Southwest Forestry University, Kunming 650224, China; yxy1999@swfu.edu.cn (C.C.); yintuo@swfu.edu.cn (T.Y.); yiwangbaoyq@swfu.edu.cn (P.T.); zhaoke@swfu.edu.cn (K.Z.); 2 Key Laboratory for Forest Resources Conservation and Utilization in the Southwest Mountains of China, Ministry of Education, Southwest Forestry University, Kunming 650224, China; lixulin@swfu.edu.cn (X.L.); wenke@swfu.edu.cn (K.W.); 3 Yunnan Agricultural Technology Extension Station, Kunming 650224, China; ranhen@163.com; 4 Institute of Tropical and Subtropical Economic Crops, Yunnan Academy of Agricultural Sciences, Baoshan 678000, China

**Keywords:** Orah mandarin, bHLH transcription factor, transcriptome, low-temperature stress, expression pattern

## Abstract

As the second largest family of transcription factors (TFs) in plants, basic helix–loop–helices (bHLHs) play key roles in regulating plant growth and development and responding to environmental stress. As the fastest growing *Citrus* variety in China in recent years, Orah mandarin has vital economic and nutritional value. Although a comprehensive genome-wide analysis of the bHLH TF family has been performed in many plants, a systematic study of the genes of this family has not been carried out in Orah mandarin. In this study, 114 bHLH TFs were identified in Orah mandarin via genome-wide analysis and were classified into 27 subfamilies according to the evolutionary tree. The gene expression profile revealed that five genes were significantly upregulated at 12 h and 24 h after low-temperature stress treatment. In addition, soluble sugars, soluble proteins, and proline contents increased with increasing low-temperature stress, which promoted the expression of the *CrbHLH46* gene, thus mediating the interconversion pathway of pentose and glucose to improve the cold tolerance of Orah mandarin. The results help explore the characteristics and functions of *CrbHLH* genes and provide a basis for further research on the Orah mandarin resistance to low-temperature stress.

## 1. Introduction

Citrus are small tree plants of the Rutaceae family, the genus Citrus, the largest genus in the Rutaceae family. It not only contains a variety of vitamins and minerals but is also closely related to the health of bones, the cardiovascular system, and the immune system; thus, it is known as “the whole body is treasure” [1]. As of 2022, China’s Citrus planting area has more than three million hectares, with an output of more than 60 million tons [2]. Today, China’s Citrus production accounts for nearly one-third of the world’s Citrus production [3], with considerable economic value and market development prospects. In recent years, China’s long-term artificial cultivation has produced some very popular *Citrus* varieties, such as the regular sweet orange, Orah mandarin (*Citrus reticulata* cv. Orah), honey orange, and grapefruit, among which the Orah mandarin variety has been the fastest growing *Citrus* variety in recent years. The cultivated area of the country has exceeded 133,333 hectares [4]. However, owing to the frequent occurrence of low-temperature and freezing damage in recent years, which has had a great impact on the production of Orah mandarin, studies on the cold tolerance of this variety have received extensive attention. The transcriptional activation and inhibition of genes, changes in metabolism, changes in membrane composition, and accumulation of osmoregulatory substances are the keys to plant resistance to cold [5].

To adapt to low temperatures, drought, and other adverse conditions, plants actively accumulate osmoregulatory substances in the body to work together to regulate the cellular osmotic pressure, thus maintaining water in the plant cell body, stabilizing the cellular structure and intercellular organization, and lowering the freezing point of the plant to adapt to changes in external environmental conditions [6]. For example, *VvBAM1* from *Vitis vinifera* overexpression affects soluble sugar levels in tomato plants by regulating starch hydrolysis, thus promoting reactive oxygen species (ROS) clearance and improving cold tolerance [7]. By spraying a biodegradable liquid film, the soluble protein content in Cabernet Sauvignon is increased to the water-holding capacity of its cells and reduces its freezing point to resist cold [8], and an increase in the proline content minimizes the water potential of cells, prevents cell dehydration, and increases cell stability to resist cold [9]; therefore, the accumulation of osmoregulatory substances is a vital way for plant cells to withstand adversity.

Transcription factors (TFs) are binding proteins that can interact specifically with cis-interacting elements. If these proteins interact with each other and other related proteins, they can inhibit or activate the transcription process to regulate the expression level of genes to adapt to the adverse external environment [10]. In recent years, a series of TFs, such as *MYB* [11], *WRKY* [12], *GRAS* [13], and *bHLH* [14], have been isolated from many plants to regulate the expression of genes related to low temperature, high salt, and drought. bHLH is a family of plant-specific transcription factors that play crucial roles in regulating plant development and stress. Its identification and expression pattern analysis are vital research approaches in functional genomics. The bHLH TF family is named after its highly conserved alkaline/helix–loop–helix domain [15], which is typically composed of 50–60 amino acids and forms two functionally distinct parts, namely, the alkaline region at the N-terminus and the HLH region at the C-terminus [16]. The bHLH domain at the N-terminus is critical for DNA binding. The C-terminal HLH region consists of two α-helices separated by a ring of variable length, which enables the formation of homodimers and heterodimers between proteins [17]. The bHLH TF family is the second largest family of TFs in plants [18]. Currently, genome-wide identification and analysis have been performed on *Arabidopsis thaliana* [19], *Malus* [20], *Solanum lycopersicum* [21], and other plants, and functional studies have shown that bHLH TFs are involved in the regulation of plant growth and development and the response to environmental stress. For example, *PebHLH35* from *Populus euphratica* acts as a positive regulator of the drought stress response by regulating the stomatal density, pore size, and photosynthesis of plants [22]. The ectopic expression of *PtbHLH173* may lead to early flowering in *Arabidopsis* [23]. *MdbHLH3* enhances the cold resistance of apples by regulating the increase in anthocyanin accumulation at low temperatures [24]. Low-temperature stress significantly induces *FtbHLH2* expression in transgenic buckwheat seedlings, and the expression of *FtbHLH2* increases the cold tolerance of transgenic *Arabidopsis* plants [25]. In wheat, *TabHLH1* can regulate the ABA-mediated stress tolerance pathway to improve plant adaptability to drought and salt stresses [26]. In blueberry fruit (*Vaccinium* spp.), *VcbHLHL1* stimulates anthocyanin accumulation and pigment development by interacting with *VcMYBL1* and *VcWDL2* [27]. In rice, *OsbHLH148* and *OsbHLH006* respond to drought stress through the jasmonic acid signaling pathway [28]. The above studies indicate that bHLH TFs, as transcriptional activators or inhibitors, play crucial biological functions in the growth and development of different species in response to stress. However, to date, no relevant studies on the family of bHLH TFs of Orah mandarin have been reported. Therefore, mining Orah mandarin bHLH TFs in response to low-temperature stress can lay the foundation for the functional analysis of cold resistance in Orah mandarin.

The purpose of this study was to comprehensively analyze bHLH family members in the whole genome of Orah mandarin and to identify and analyze their physical and chemical properties, perform evolutionary analysis, determine their gene structure and conserved motif, analyze their cis-acting regulatory elements, and explore the changes in osmoregulatory substances and their response to low-temperature stress. This study provides a foundation for understanding the evolution and biological functions of the bHLH TF family of Orah mandarin. It supplies a basis for further research on resistance breeding in Orah mandarin under low-temperature stress.

## 2. Results

### 2.1. Subsection Identification and Physicochemical Property Analysis of the bHLH TF Family

With the protein sequences of the *Arabidopsis* bHLH TF family as a reference, BLAST-2.13.0 software and hidden Markov model (HMM) searches were carried out on the genomic protein data of Orah mandarin. Then, the genes of its bHLH TF family were subsequently predicted by CDD and Pfam, incomplete or nonexistent structural domains were removed, and a total of 114 *bHLH* genes were identified and named *CrbHLH1*-*CrbHLH114* based on the results of their evolutionary analyses. The number of amino acids, molecular weights (MWs), and isoelectric points (pIs) were calculated based on the protein sequence of each identified protein. The number of predicted amino acids was 54 (*CrbHLH5*)–1340 (*CrbHLH88*). The molecular weights were 6209.97 kDa (*CrbHLH5*)–499898.70kDa (*CrbHLH74*). The theoretical pI value was 4.70 (*CrbHLH48*)–10.13 (*CrbHLH70*), and the instability index was 35.94 (*CrbHLH42*)–84.39 (*CrbHLH80*). In particular, the only one less than 40 was *CrbHLH42*, suggesting that all the proteins, except for this one, were unstable. In terms of amino acid composition, the average aliphatic index was 74.87, which favors the thermal stability of globular proteins, and the hydrophilicity ranged from −0.945 (*CrbHLH100*) to 0.266 (*CrbHLH109*), which are hydrophilic proteins (for details, see Table A1).

### 2.2. Evolutionary Analysis

**Figure 1:**
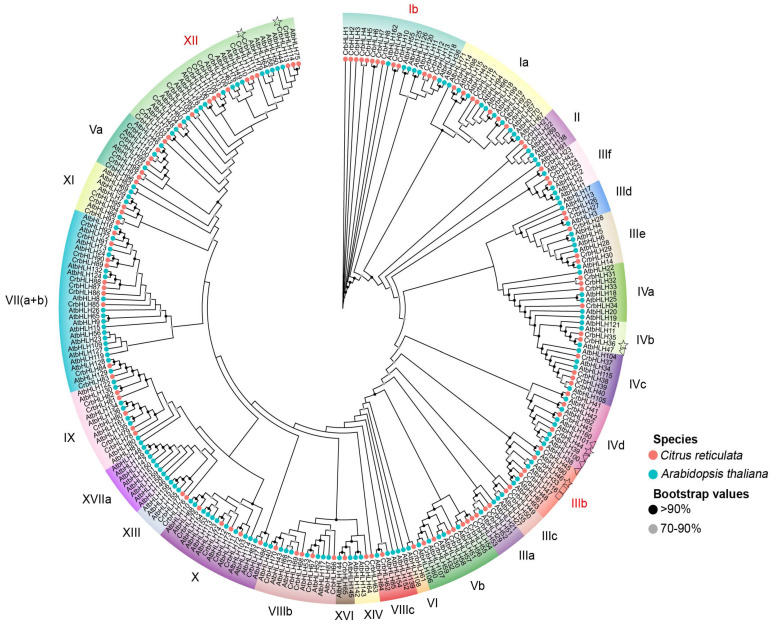
Evolutionary analysis tree of bHLH TF family in *Arabidopsis* and Orah mandarin. Subfamilies marked in red indicate subfamilies in *Arabidopsis* that have been proven involved in abiotic stress. Some TFs with different functions are labeled with various shapes. The pentagrams mark the differentially expressed genes. In *Arabidopsis*, the genes involved in iron deficiency stress are triangular, and the genes involved in low-temperature stress are square.

To understand the evolutionary relationship between the bHLH TF family of Orah mandarin and other bHLH TF family genes, we constructed an evolutionary tree via multisequence comparisons of 114 bHLH protein sequences of Orah mandarin and *Arabidopsis*. The ML methods of MEGA11.0 and IQtree were used to build the evolutionary tree, and the results revealed that the evolutionary trees constructed by the two methods had consistent tree topology (Figure 1). Based on evolutionary tree results and the classification of *AtbHLHs* in *Arabidopsis* [19], the bHLH TF family can be divided into 27 subfamilies. Different subfamilies have certain preferences in coping with various biological or abiotic stresses, and members from the same evolutionary branch often have highly similar stress functions [29]. Among them, CrbHLH constitutes the largest number in subfamily XII, which contains 30 family members, including 14 CrbHLH TFs and 16 AtbHLH TFs. The VI subfamily has the smallest number of genes, containing only one CrbHLH TF and one AtbHLH TF, whereas the XVI subfamily also contains only one CrbHLH TF but two AtbHLHs.

### 2.3. Analysis of Conserved Motifs and Gene Structure

**Figure 2:**
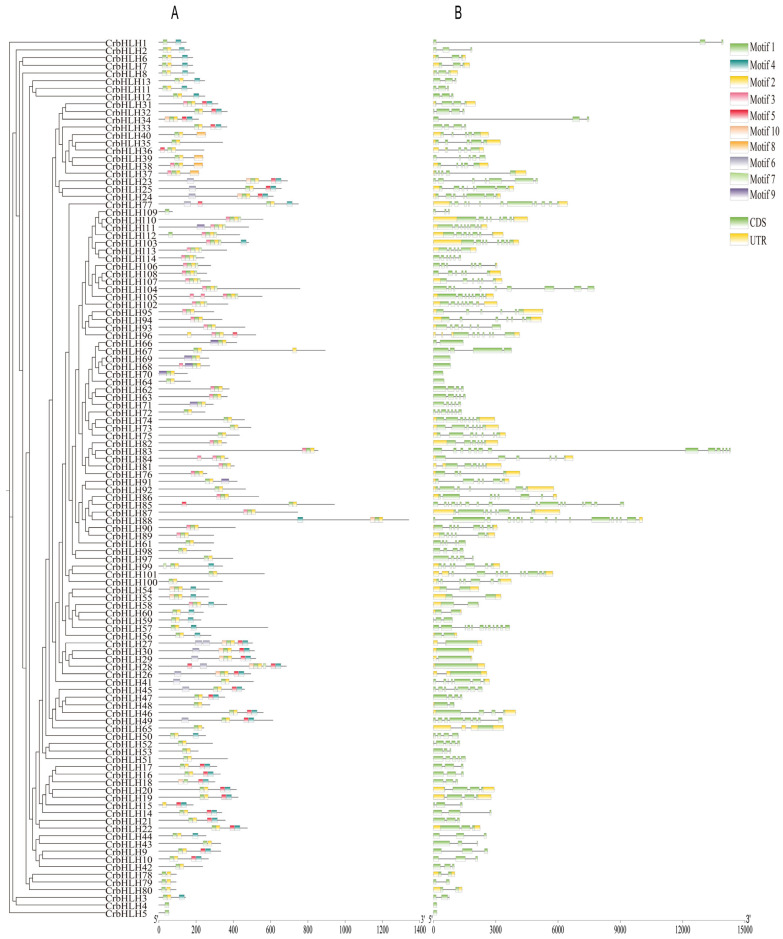
Evolutionary relationships, conserved motifs (**A**), and gene structure (**B**).

Conserved modules are crucial in mediating protein interactions to control cell processes [30]. Therefore, to further study the sequence characteristics of the CrbHLH protein, this study used the MEME online tool to analyze ten conserved modules of this protein (Figure 2A). The results revealed that most CrbHLH TFs have two or more conserved motifs. Motif 1 and Motif 2 are highly conserved in almost every CrbHLH protein. However, the same motifs have different positions in different protein sequences, which may be related to the structure and function of this protein. Even members of the same subfamily have certain differences in their motif distributions. For example, Motif 2 is not found in CrbHLH1 in subfamily Ia, which may be related to the differences between genes of transcription factor families under certain conditions. In addition, each group presented some degree of motif specificity, such as Motif 6 in only the IIIf and IIIe subfamilies and Motif 8 in only the IVc subfamily.

Gene structure plays a key role in the evolution of gene families, so studying exon/intron patterns contributes to the understanding of evolutionary relationships among members of the *bHLH* TF family in Orah mandarin [31]. Therefore, we sequentially aligned 114 *CrbHLH* genes and analyzed their gene structure based on genome annotation files (Figure 2B). Genes in the same subfamily contain similar numbers of exons and introns, and their locations are relatively conserved. The number of exons varies from 1 to 13, with relatively large numbers in subfamilies IIIb, X, VIII (a + b), X, and XI. Most *CrbHLH* genes contain more than 2 introns, but eight genes contain no introns, and *CrbHLH85* has 16 introns, the highest number. However, overall, the intron/exon distribution patterns within the *CrbHLH* subfamily are similar.

### 2.4. Analysis of Cis-Acting Elements

**Figure 3:**
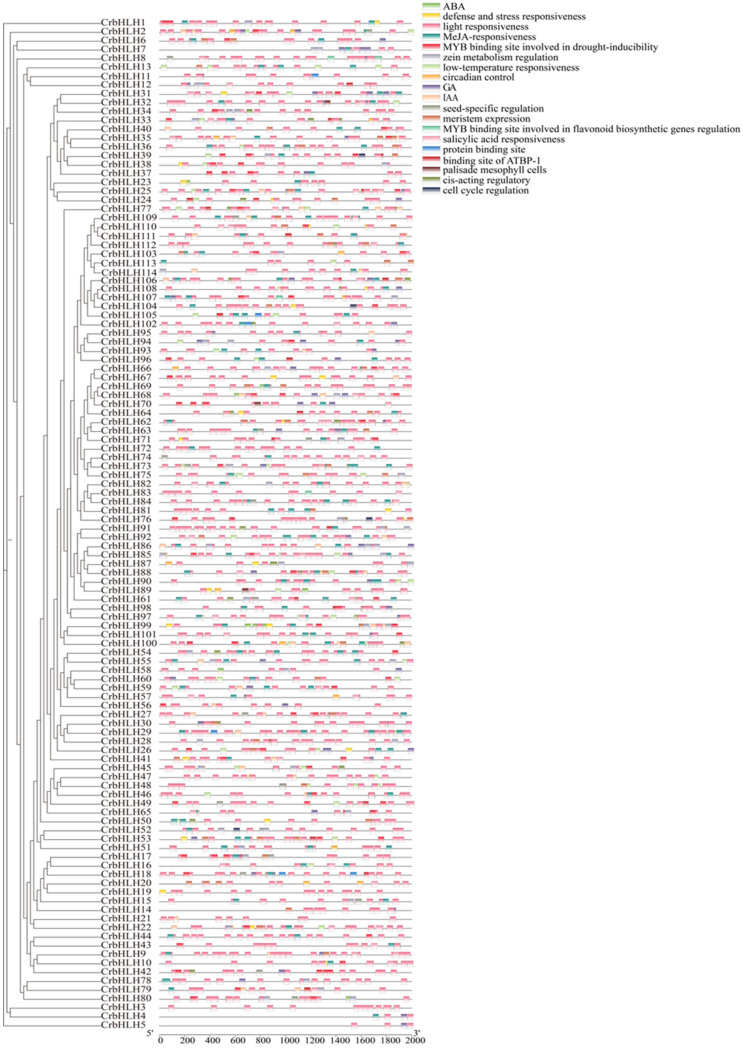
Distribution of cis-acting elements in the upstream 2000 bp sequence of 114 *CrbHLH* TF family genes. Different cis-acting elements are represented by different colors.

To further elucidate the potential functions of members of the *CrbHLH* TF family, cis-acting elements in the upstream 2000 bp region of the promoter were analyzed. The results revealed that the promoter region of *CrbHLH* contained many cis-responsive components (Figure 3), of which light-responsive components were distributed on almost every gene. We hypothesized that members of the gene family should be able to respond positively to light-related responses. However, hormone-related cis-responsive components are inferior to only light-responsive components. These include MeJA, ABA, GA, IAA, and salicylic acid. Moreover, we identified many cis-acting elements involved in the abiotic stress response, which are related to environmental stresses, such as low temperature and drought, as cis-acting elements. Other cis-acting elements were less distributed, such as the binding sites of ATBP-1 response elements, MYB binding sites, and seed-specific regulatory elements, among which the MYB binding sites were distributed in the promoters of all *CrbHLH* TF family genes on average. In general, most of the homeopathic elements of promoters are involved in abiotic stress and hormone responses, which can regulate gene expression and substance metabolism in plants to produce plant stress resistance and improve the adaptability of Orah mandarin under stressful environments.

### 2.5. GO Function Annotation of the CrbHLH TF Family

**Figure 4:**
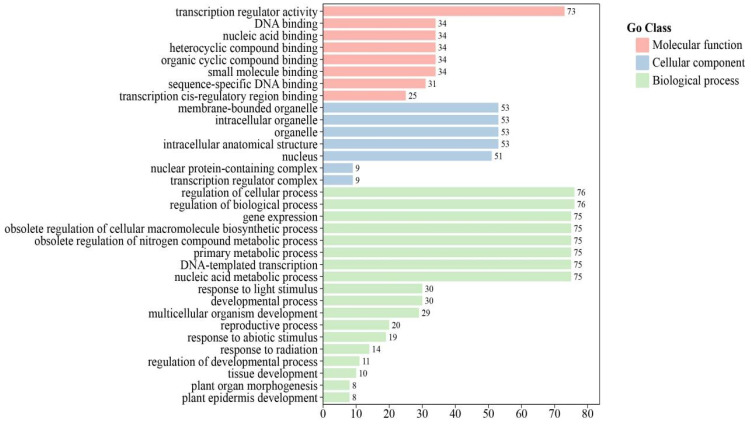
GO enrichment analysis of 114 *CrbHLH* TFs under low-temperature stress.

To further elucidate the biological functions of the 114 *CrbHLH* TF family genes, the EggNOG-MAPPER database was used in this study to annotate the gene functions of their gene proteins, and the online tool ChiPlot was used for mapping and visualization (Figure 4). The annotations revealed that the results were assigned to three broad categories: molecular function (MF), biological process (BP), and cellular component (CC). With respect to molecular functions, the enrichment of *CrbHLH* genes was greater for nucleic acid binding and organic ring compound binding. However, in terms of cell components, the concentration of *CrbHLH* genes was higher in cell components and nuclei. In particular, most genes were enriched in biological processes, and these biological processes play crucial roles in the regulation of the *CrbHLH* TF family. Among them, *CrbHLH* genes are more abundant in terms of synthesis and metabolism, growth and development, and environmental stimulus response, among which are environmental stimulus responses such as abiotic stress response and light response. Growth and development processes, such as plant organ development, reproductive system regulation, and tissue development, suggest that *CrbHLH* TF family genes may adapt to different environments by regulating different parts of plants.

### 2.6. Analysis of Protein Network Interactions of the bHLH Transcription Factor Family of Orah Mandarin

**Figure 5:**
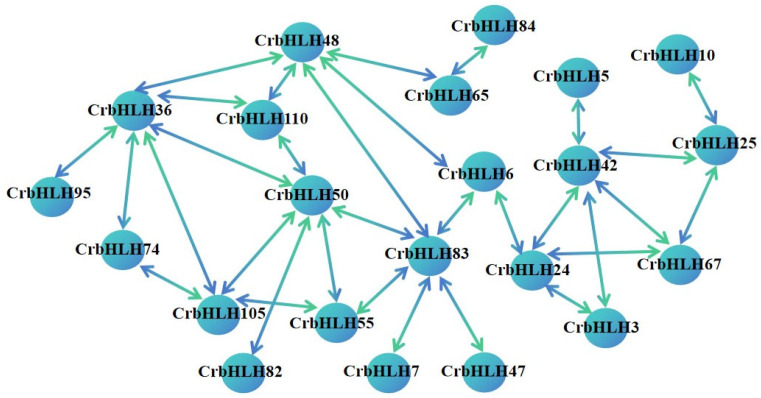
The PPI interaction diagram.

Network interaction relationship analysis of 114 CrbHLH proteins revealed that 22 interact with each other (Figure 5), but 92 proteins could not form interacting relationships, indicating that 22 proteins play vital roles. Among them, five proteins belonged to subfamily Ib, two proteins belonged to subfamily IIIb, and one protein belonged to subfamily XII. This family of proteins is known to be involved in the abiotic stress response [32,33,34], and other proteins are distributed in IIIf, VII(a + b), VIIIb, IVb, and other subfamilies.

### 2.7. Analysis of Phenotypic and Osmoregulatory Substance Content Changes in Orah Mandarin Under Low-Temperature Stress

**Figure 6:**
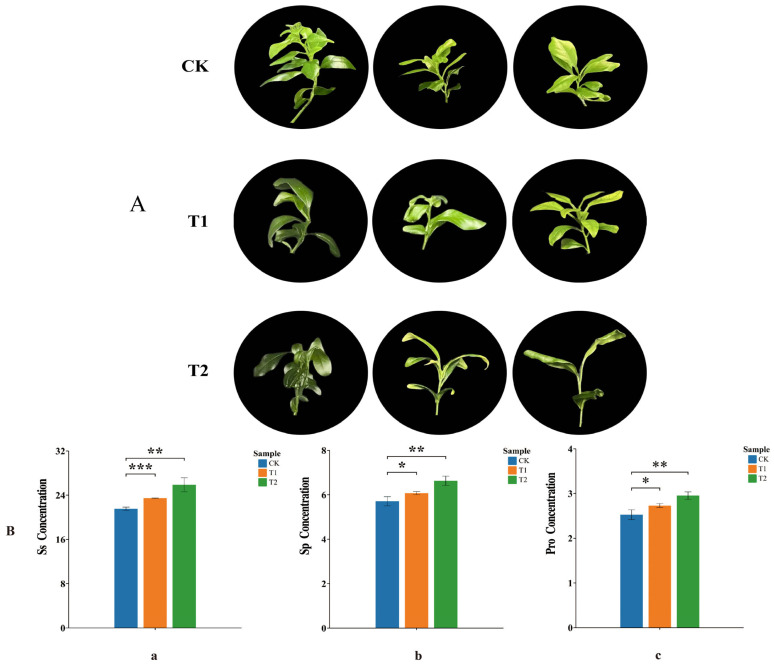
(**A**) Phenotypic observations of Orah mandarin under low-temperature stress; CK is a control group grown at 25 °C; T1, treatment for 12 h; T2, treatment for 24 h. (**B**) Os content of Orah mandarin under low-temperature stress: (**a**) is soluble sugar (Ss), (**b**) is soluble protein (Sp), and (**c**) is proline (Pro). * is *p* < 0.05, ** is *p* < 0.01, *** is *p* < 0.001.

Osmoregulation is a crucial way for plant cells to resist cold. Plants accumulate osmoregulatory substances to jointly regulate cell osmotic pressure to prevent excessive water loss and resist cold [6]. Therefore, we analyzed the phenotypic changes and physiological indexes of Orah mandarin. Compared with the control, salt stress severely inhibited the growth of Orah mandarin seedlings, resulting in curling and even falling of their leaves (Figure 6A), indicating that the growth, development, and physiological metabolism of plants are severely impaired under low-temperature stress and that plants adapt to environmental changes. The contents of soluble sugars, soluble protein, and proline increased with increasing duration of low-temperature stress, and their expression was significant (Figure 6B). Therefore, we hypothesized that when Orah mandarin is subjected to low-temperature stress, the cells in its body complete its regulatory process by changing its leaf morphology and synthesizing osmoregulatory substances, thus affecting its gene expression to increase cold tolerance.

### 2.8. Analysis of the Expression Patterns of the CrbHLH TF Family in Orah Mandarin Under Low-Temperature Stress

**Figure 7:**
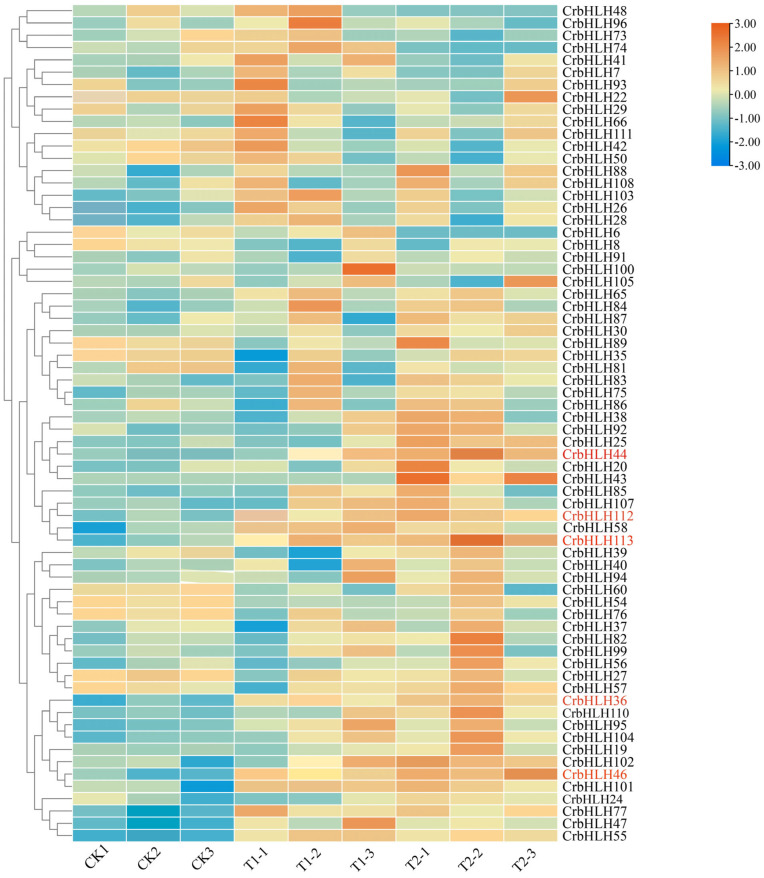
Heatmaps of the expression profiles of 68 *CrbHLH* genes under low-temperature stress. Different colored boxes represent different log2 (FPKM) values, with CK for the control group and T for the treatment group. The genes marked in red are significantly expressed genes.

The expression patterns of genes can reflect the functional characteristics of genes to a certain extent [35]. Therefore, to understand the expression patterns of *CrbHLH* genes in Orah mandarin under low-temperature stress, we analyzed the transcriptome data of Orah mandarin after low-temperature stress. The results revealed that under low-temperature stress, 49 genes were upregulated, and 17 were downregulated in T1, whereas 50 genes were upregulated and 17 were downregulated in T2. Compared with those in the control group, there were individual genes that were significantly expressed only in T1 or T2, such as *CrbHLH43* and *CrbHLH102*. Five genes that were significantly expressed in both T1 and T2 (Figure 7) were *CrbHLH36*, *CrbHLH44*, *CrbHLH46*, *CrbHLH112*, and *CrbHLH113*, all of which were upregulated, indicating that the *CrbHLH* gene can positively regulate the damage caused by low-temperature stress on Orah mandarin to improve the cold resistance of Orah mandarin. We speculate that the *CrbHLH* gene is a vital gene affecting Orah mandarin under low-temperature stress.

### 2.9. qRT-PCR

**Figure 8:**
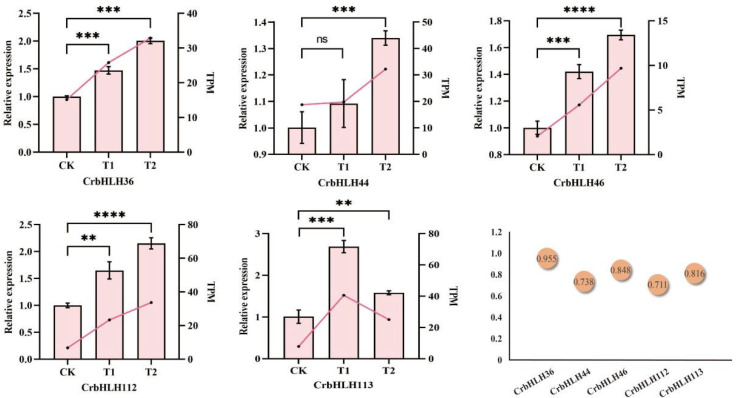
qRT-PCR validation of 5 *CrbHLH* significantly expressed genes under low-temperature stress. The pink line segments connect TPM values, the rectangular bars indicate the relative expression values, and the orange circle values indicate the correlation coefficients between the relative expression and TPM, ** is *p* < 0.01, *** is *p* < 0.001, **** is *p* < 0.0001, ns is not significant, and all the data were averaged from the means of three biological replicates.

To further verify the candidate low-temperature response genes, we selected five genes that were significantly expressed in T1 and T2, namely *CrbHLH36*, *CrbHLH44*, *CrbHLH46*, *CrbHLH112*, and *CrbHLH113*, and verified their expression via qRT-PCR analysis. The results revealed that these genes presented similar expression profiles under low-temperature stress, all of which were upregulated, and the expression trend was consistent with the results of the RNA-seq analysis. The correlation coefficients were all greater than 0.7 (Figure 8), indicating that these genes may play crucial synergistic or antagonistic regulatory roles under low-temperature stress. The T2 values of some genes were slightly lower than those of T1. We speculated that, when low-temperature stress was applied for a certain period, gene function was affected to some extent.

## 3. Discussion

The bHLH TF family is the second largest family of TFs in plants [18] and is widely distributed in eukaryotes. Numerous studies have shown that bHLH TFs play crucial roles in plant physiological processes, such as plant growth and development [36]; abiotic stress responses, such as low temperature, drought, and salt stress [37]; and anthocyanin synthesis [38]. However, there are few reports on the function of the bHLH TF family of Orah mandarin. Therefore, in this study, the members of the bHLH TF family in the genome of Orah mandarin were identified, and the sequence information, characteristics, and expression patterns of each member under low-temperature stress were analyzed in detail, which can lay a foundation for the genetic improvement of the cold resistance of Orah mandarin.

In this study, a total of 114 *CrbHLH* genes were identified, more than in limonash (75) [17], ginkgo (85) [39], and capsicum (107) [40] and less than in cucumber (142) [41], passion fruit (138) [42], and azaleas (145) [43]. The variation in the number of *bHLH* genes among different species may be attributed to events such as genome/gene duplication or differences in genome size [44]. According to the evolutionary analysis, most of the CrbHLH TFs formed good clusters with those of *Arabidopsis*, suggesting that these CrbHLH may have had some fundamental biological functions during the long-term evolution of Orah mandarin. Among them, there are more CrbHLH TFs in the Ib and XII subfamilies. Different TFs within the same subfamily may share a common evolutionary origin and may have similar physiological functions [45]. For example, the ZmbHLH XIV subfamily is closely related to Group VII of the PIF family in *Arabidopsis* and plays vital roles in light signal transduction, seed germination, and shade avoidance [46]; CpbHLH118, CpbHLH66, and CpbHLH96 in *Chimonanthus praecox* cluster with AtbHLH1, AtbHLH2, AtbHLH12, and AtbHLH42 in the IIIf subfamily, which are associated with anthocyanin and proanthocyanidin biosynthesis, trichome formation, and root hair patterning [47]. Research has confirmed that the Ib and XII subfamilies are related to the abiotic stress response [33,34], indicating that they have certain functional similarities, and this result has been further demonstrated via protein interaction network analysis. Conserved motif analysis revealed that similar genes were clustered in the same subfamily and that there were multiple motifs in specific proteins, which meant that they might have specific functions, among which Motif 1 and Motif 2 were present in most proteins, reflecting their importance to CrbHLH proteins. This result is consistent with the phenotype bHLH TFs in plants such as *Quercus mongolensis* [48], *Hordeum vulgare* [49], and maize [46]. The gene structure shows that the number of exons of *CrbHLH* TFs varies from 1 to 13. Most *CrbHLH* genes contain more than two introns. However, eight genes contain no introns, significantly different from other *CrbHLH* genes, indicating that the exon–intron structure has undergone insertion or deletion during evolution [50]. In conclusion, the proteins clustered in the same subfamily in the evolutionary tree have similar conserved motifs and gene structures. These findings further indicate that genes with similar evolutionary processes may also have similar functions, thus helping to screen *bHLH* genes with similar functions.

**Figure 9:**
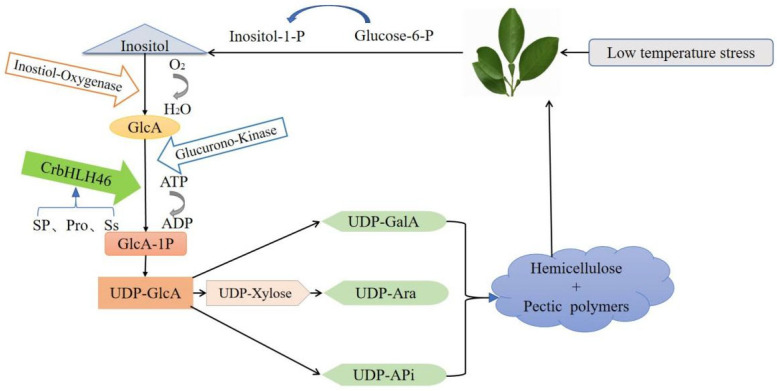
Pathway diagram of mutual conversion between pentose and glucose in KEGG-annotated plants induced by *CrbHLH46* induced by osmoregulatory substances under low-temperature stress; Ss represents soluble sugars, Sp represents soluble protein, and Pro represents proline.

Cis-acting elements play vital regulatory roles in the transcription of neighboring genes. In this study, many plant growth-related response elements, most of which are photoresponsive cis-acting elements, hormone-related cis-acting elements, and environmental stress response elements, were detected in the promoter region of the *CrbHLH* TF. This finding is consistent with the results that many plants, such as poplar, grape, and rape, are regulated by these cis-acting elements in response to abiotic stress, and regulate gene expression and material metabolism in plants at the hormone level and signal transduction level to produce stress resistance, thus improving the adaptability of plants under stressful environments [51,52,53]. When plants are stressed, they respond through corresponding life activities to maintain normal intracellular activities, among which genes whose expression significantly differs may participate in the corresponding response pathway. In this study, the expression pattern of the *CrbHLH* gene under low-temperature stress was analyzed, and the results revealed that, compared with those in the control group, five genes belonging to the IVb, IVd, IIIb, and XII subfamilies were significantly upregulated in T1 and T2 at the same time. Among them, the *CrbHLH112* and *CrbHLH113* genes belong to the same subfamily XII, which is related to the abiotic stress response [34], and *CrbHLH36* and *CrbHLH44* belong to the same branch as *AtbHLH47* and *AtbHLH100* genes in Arabidopsis, which have been verified to be stressed by iron deficiency [54]. Members from the same evolutionary branch tend to have highly similar stress functions [29], so we speculate they have relatively identical functions. Notably, the significantly different gene *CrbHLH46* belongs to the IIIb subfamily, which has been identified as participating in the abiotic stress response and belongs to the same branch as *AtbHLH33* and *AtbHLH116* in *Arabidopsis*, which have been verified to respond to low-temperature stress. The KEGG results revealed that *CrbHLH46* mediated the mutual conversion pathway between pentose and glucose. Moreover, the contents of osmotic regulatory substances, such as soluble sugars and proline, increased during this process (Figure 9). When plants are subjected to low-temperature stress, they will biosynthesize osmoregulatory substances to promote the expression of related genes and form complex networks with stress signaling pathways, thereby regulating plant metabolism and increasing the stability of the cell wall, which is composed of a large amount of pectin and hemicellulose, to resist cold [55,56]. Therefore, the mutual conversion pathway between pentose and glucose is crucial in plant resistance to cold [57]. Studies have shown that the UDP-GlcA in this pathway provides approximately 50% of the cell wall biomass, is a key metabolite for the synthesis of pectin and hemicellulose in the nucleotide sugar exchange process [58], and is a direct precursor of cell wall biosynthesis [59]. When plants are subjected to low-temperature stress, *CrbHLH46* is differentially expressed under the induction of osmoregulatory substances such as soluble sugars and proline and may positively regulate the action of genes such as MIOX, USP, and others on GlcA to produce UDP-GlcA to increase hemicellulose and pectin contents, thus increasing the cold tolerance of plants [59,60,61,62].

## 4. Materials and Methods

### 4.1. Identification and Physicochemical Properties of the bHLH TF Family

From the Citrus Pan-genome to Breeding Database (http://citrus.hzau.edu.cn/ accessed on 5 October 2024), we downloaded genome data, protein sequences, and comments in files, and *Arabidopsis* bHLH protein sequence information from TAIR [63] (https://www.arabidopsis.org/ accessed on 5 October 2024). Based on the downloaded results, the hmmsearch program of HMMER 3.0 software was used to search the conserved domain of the bHLH TF family (ID: PF00010) in the genomic protein database of Orah mandarin and protein sequence numbers with E values < 1 × 10^−5^ were screened from the results [64]. Second, BLAST-2.13.0 software was used to blast the genomic protein data of Orah mandarin based on the AtbHLH protein sequence, and the sequences with high homology (E value < 1 × 10^−5^) with the AtbHLH TF family in the genomic protein data were screened out, and the above steps were repeated [65]. Finally, the sequence numbers obtained in the above two steps were combined, and the TBtools software (version 1.113) was used to extract the corresponding sequence from the genomic protein data and save it as a *.fast file [66]. Using NCBI conserved domain database (CDD) tools (https://www.ncbi.nlm.nih.gov/Structure/cdd/wrpsb.cgi accessed on 10 October 2024) and the online software PFAM (http://pfam.xfam.org/search#tabview=tab1 accessed on 10 October 2024), the existence of bHLH domains was confirmed, and nonexistent or incomplete domain sequences were removed. Finally, the members of the bHLH TF family of tangerines were identified [67]. The ExPASy website (https://web.ExPASy.org/ accessed on 10 October 2024) was used to predict the length, relative molecular weight, theoretical isoelectric point, hydrophilicity, and other physicochemical properties of proteins encoded by the CrbHLH TF family [68].

### 4.2. Evolutionary Analysis

The identified bHLH protein sequences of Orah mandarin and *Arabidopsis* were combined, sequences alignment was performed via MAFFT software [69] and pruning via TBtools software (version 1.113) [66], and the obtained results were constructed via the MEGA11.0 and maximum likelihood (ML) method of IQtree [70]. Chiplot (https://www.chiplot.online/ accessed on 1 November 2024) online software was used for evolutionary tree visualization [71]. Finally, according to the grouping of *Arabidopsis* bHLH proteins, 114 bHLH proteins were renamed and grouped according to the *CrbHLH*+ serial number [19].

### 4.3. Analysis of Gene Structure and Conserved Motifs

In accordance with the genome annotation file (GFF3) of Orah mandarin, TBtools software (version 1.113) was used to analyze the gene structure of each gene [57]. MEME (http://meme-suite.org/ accessed on 25 November 2024) was used to analyze the conserved motif of the bHLH protein to analyze the differences among the members of the CrbHLH TF family. Finally, TBtools software (version 1.113) was used to visualize the above results [72].

### 4.4. Analysis of Cis-Acting Elements

For identification, the gene ID was obtained, and the base sequence 2000 bp upstream of each gene was extracted from the genome annotation file (GFF3) via TBtools software (version 1.113). The online analysis software PlantCARE (https://bioinformatics.psb.ugent.be/webtools/plantcare/html/ accessed on 5 December 2024) was subsequently used to conveniently perform promoter element analysis [73].

### 4.5. GO Enrichment Analysis

The protein sequences of 114 *CrbHLH* TFs were submitted to the EggNOG-MAPPER database (http://eggnog-mapper.embl.de/ accessed on 10 December 2024) for gene function annotation [74], and the obtained results were then analyzed for GO function enrichment via TBtools software (version 1.113). The online tool ChiPlot (https://www.chiplot.online/ accessed on 12 December 2024) was used for mapping and visualization [71].

### 4.6. Analysis of Protein Network Interactions

The STRING protein–protein interaction (PPI) database (http://string-db.org/ accessed on 25 December 2024), which records the protein interactions of most species, was used. A network interaction diagram of 114 bHLH proteins in Orah mandarin was constructed and then beautified, and a single protein that could not form an interaction relationship no longer appeared [46].

### 4.7. Analysis of CrbHLH Expression Patterns in Orah Mandarin in Response to Low-Temperature Stress

In this study, the seeds of Yunnan Xinping Orah mandarin were selected, and the inoculants were obtained via tissue culture. When the seeds were subjected to low-temperature stress, the temperature that had an obvious influence on growth was 4 °C. In an artificial climate chamber with a light period of 16 h/8 h of darkness, 4 °C was selected for low-temperature stress treatment, and samples were taken at 12 h and 24 h, denoted as T1 and T2, compared with 25 °C in the same environment, denoted as CK. Three biological replicates were set for each treatment, and the treated samples were stored in a liquid nitrogen environment at −80 °C. Then, the samples were sent to Baipu Bioscience and Technology Company to detect and extract RNA with advanced equipment in molecular biology. After passing quality inspection, the qualified libraries were sequenced by a high-throughput sequencing platform with PE150 mode.

### 4.8. Determination of Soluble Sugar Content Under Low-Temperature Stress

Approximately 0.1 g of the leaves were weighed, ground into a powder with liquid nitrogen, and then ground into a homogenate with 1 mL of distilled water. The sample was heated in a water bath at 95 °C for 10 min, cooled, and centrifuged at room temperature at 8000× *g* for 10 min. Then, 50 μL of cooled supernatant was taken, and 6 mol/L HCl was added to the sample at 80 °C for 30 min. Then, 10 μL of 6 mol/L NaOH and 30 μL of distilled water were added, 50 μL was added to a 1.5 mL centrifuge tube, 50 μL of DNS reagent was added to the tube, the mixture was incubated in a boiling water bath for 5 min, the mixture was cooled to room temperature, 200 μL of distilled water was added, and 200 μL was mixed at 540 nm to determine its absorbance value.

### 4.9. Determination of Soluble Protein Content Under Low-Temperature Stress

The leaves weighed approximately 0.1 g, were ground into a powder with liquid nitrogen, ground into a homogenate with 1 mL of distilled water at 8000 g, and centrifuged at 25 °C for 10 min. After completion, 20 μL of the supernatant was added to a 96-well plate, 200 μL of Coomassie bright blue G-250 solution was added, mixed well, and the absorption value at 595 nm was measured.

### 4.10. Determination of the Proline Content Under Low-Temperature Stress

Approximately 0.2 g of the leaves were ground into a powder with liquid nitrogen and then put into a large test tube with a plug. Then, 5 mL of 3% sulfosalicylic acid was added to the tube, which was soaked in a boiling water bath for 10 min after plugging, 2 mL of the supernatant was absorbed into the test tube after cooling, and 2 mL of ice acetic acid and 3 mL of acid indanhydrin color developing solution were added. The mixture was heated in a boiling water bath for 40 min for color development. The mixture was removed and allowed to cool to room temperature, 5 mL of toluene was added, the mixture was shaken fully to extract red material, the mixture was allowed to stand stratified and absorb the toluene layer, and the absorbance was determined with a 520 nm spectrophotometer.

### 4.11. Analysis of the Expression Patterns of the CrbHLH TF Family Under Low-Temperature Stress

To avoid problems such as low-quality sequences and joint contamination in the transcriptome data, we used Trim Galore and Trimmomatic software to remove adapters and filter low-quality data, and employed FastaQC to check data quality, ultimately ensuring that all the transcriptome data had q values greater than 30 [71]. The reads of the qualified transcripts were located to the reference genome via HISAT2 [75], and the reads with the reference genome were counted via the features toolkit from Rsubread software. The fragments per kilobase of exon model per million mapped fragments (FPKM) value of each gene was calculated, and, finally, the expression of each gene was quantified [76]. To make the gene expression estimated by different experiments comparable, we chose the most common gene expression normalization method to calculate the transcripts per million (TPM) value of each gene, which could integrate the influence of sequencing depth and gene length on the read count and normalize the expression of each gene. Based on the log2 (TPM + 1) value, TBtools was used to map the expression profiles of *CrbHLH* TF family members under low-temperature stress. In this study, DESeq2 software was used for differential expression gene analysis [77], and a fold change ≥1.5 and false discovery rate (FDR) < 0.05 were used as screening criteria. Eligible genes were differentially expressed genes (DEGs).

### 4.12. Verification via qRT-PCR Under Low-Temperature Stress

To verify the accuracy of the RNA-Seq data, based on the fold change values, we selected 5 *CrbHLH* genes, namely *CrbHLH36, CrbHLH44, CrbHLH46, CrbHLH112, and CrbHLH113* [63]. According to the instructions of the DP43 Kit, the FastKing RT Kit was used to extract Orah mandarin and synthesize cDNA, and qRT-PCR was performed according to previously reported procedures [71]. Real-time PCR was performed with an ABI7500 instrument, and the reaction system was established as follows: 10 µL of power qPCR mix, 0.5 µL of forward primer and reverse primer, 1 µL of diluted cDNA template, and, finally, 20 µL of RNase-free ddH_2_O. The reaction procedure was as follows: 95 °C for 10 min, 95 °C for 10 s, and 60 °C for 40 s for 42 cycles. We used an internal reference gene for the experiment. *CrActin* (MSYJ123420.2) was selected as the internal reference gene to normalize the data [78,79], three biological replicates were used for each gene, and the relative expression of the genes was calculated via the 2^−ΔΔCt^ method [80]. SPSS (IBM Statistics 26) was used for statistical analysis, and an independent sample t-test was used to calculate the *p* value [81]. All primers used are listed in Table 1.

## 5. Conclusions

In this study, the bHLH TF family of Orah mandarin was systematically analyzed. In total, 114 CrbHLH TFs were identified in the genome of Orah mandarin, which were divided into 27 subfamilies via evolutionary analysis. A combination of gene structure and conserved motif analysis revealed that members of the same subgroup presented similar characteristics. In addition, gene expression profile analysis revealed that five genes, all of which were upregulated, were significantly different between T1 and T2. Among these genes whose expression significantly differed, the *CrbHLH46* gene was unique. It positively regulates GlcA to produce UDP-GlcA under the action of osmoregulatory substances to increase cell wall resistance, thereby making plants resistant to cold. This study provides a theoretical basis for breeding cold-resistant varieties of Orah mandarin. These findings lay the foundation for improving the cold tolerance of Orah mandarin through genetic engineering technology in practice and can be used to explore more features and functions of *CrbHLH* genes in the future.

## Figures and Tables

**Table 1:** Information on the primers.

Gene	Sequence (5′–3′)	
*MSYJ123420.2 (Actin)*	F	ACTGAGCACCACATTCCCATACA
	R	GCAAGTCATAACTATTGGAGCCG
*MSYJ232700.4*	F	GAGGAGACGCAGGAAGAAGCTC
	R	GTCAATGCTGAACCAGGAGGG
*MSYJ208070.1*	F	TTCCAAGCCTTGGGTGGC
	R	GCTCGCTGGCATTGTGATAGA
*MSYJ280900.2*	F	GCAAGGAGGGGTCAAGCAAC
	R	CTTGTCACAGCCAGGAACAAGC
*MSYJ174650.1*	F	CAACAAGTGAAGCCCGACCC
	R	ATTCAAAGCAGCCACTGACACC
*MSYJ037560.2*	F	TGCAGAGCTAAAGCCGAGCAT
	R	CTTACGGTACGAACACGGGGTA

## Data Availability

All the data generated or analyzed are included in this article. Orah mandarin genome annotation files can be found at http://citrus.hzau.edu.cn/ (accessed on 5 October 2024). RNA-Seq data under low-temperature stress can be found, and the registration number is PRJNA1202851. The RNA-Seq data are publicly available at the National Center for Biotechnology Information. The other data presented in this study are available in the Appendix A. All experimental studies and experimental materials involved in this research are in full compliance with relevant institutional, national, and international guidelines and legislation.

## Appendix A

**Table A1:** Analysis of the physicochemical properties of *CrbHLH* TFs.

Sequence ID	Gene Name	Number of Amino Acids (aa)	Molecular Weight (kDa)	Theoretical pI	Instability Index	Aliphatic Index	Grand Average of Hydropathicity (GRAVY)
MSYJ191700.1	*CrbHLH1*	146	16,347.6	6.92	44.51	92.12	−0.472
MSYJ273420.1	*CrbHLH2*	164	18,394.11	6.97	48.49	93.29	−0.349
MSYJ273410.1	*CrbHLH3*	142	15,916.98	5.95	64.53	87.25	−0.436
MSYJ045200.1	*CrbHLH4*	78	6308.10	9.18	54.75	70.35	−0.726
MSYJ152660.1	*CrbHLH5*	54	6209.97	9.40	58.85	72.41	−0.785
MSYJ073010.1	*CrbHLH6*	181	20,709.95	9.22	42.80	84.53	−0.470
MSYJ231530.1	*CrbHLH7*	181	20,682.89	9.05	43.20	90.39	−0.403
MSYJ185140.7	*CrbHLH8*	189	21,165.74	6.09	45.38	74.87	−0.629
MSYJ246180.1	*CrbHLH9*	331	36,216.05	8.18	55.35	75.17	−0.495
MSYJ258050.1	*CrbHLH10*	265	29,635.65	6.98	55.38	74.72	−0.505
MSYJ210310.1	*CrbHLH11*	177	19,651.56	8.35	65.55	96.21	−0.326
MSYJ107100.1	*CrbHLH12*	246	28,324.92	6.32	71.43	79.23	−0.722
MSYJ210300.1	*CrbHLH13*	245	27,704.23	6	39.57	88.65	−0.559
MSYJ118000.1	*CrbHLH14*	337	36,863.42	5.06	59.38	85.64	−0.432
MSYJ166830.1	*CrbHLH15*	183	20,360.77	6.96	43.26	116.07	0.074
MSYJ064870.1	*CrbHLH16*	329	36,393.74	5.58	68.81	77.14	−0.469
MSYJ048500.1	*CrbHLH17*	310	34,673.10	6.84	63.69	67.94	−0.471
MSYJ229290.1	*CrbHLH18*	300	34,083.89	5.10	62.91	79.33	−0.641
MSYJ104770.2	*CrbHLH19*	424	48,007.90	5.35	57.36	77.26	−0.654
MSYJ112160.1	*CrbHLH20*	415	47,086.40	5.61	70.83	78.94	−0.551
MSYJ179400.1	*CrbHLH21*	355	39,839.61	5.62	49.24	78.85	−0.539
MSYJ107260.3	*CrbHLH22*	474	53,314.32	5.65	52.49	72.41	−0.686
MSYJ218540.1	*CrbHLH23*	689	76,292.21	4.97	64.27	77.52	−0.587
MSYJ233170.1	*CrbHLH24*	613	68,767.58	5.84	45.03	83.15	−0.466
MSYJ110120.1	*CrbHLH25*	656	73,535.07	5.18	53.80	82.21	−0.434
MSYJ111470.2	*CrbHLH26*	492	54,578.53	7.73	38.01	79.25	−0.460
MSYJ219740.1	*CrbHLH27*	502	55,604.62	6.16	46.40	73.37	−0.466
MSYJ000950.1	*CrbHLH28*	683	74,406.52	5.50	53.34	66.54	−0.613
MSYJ269980.1	*CrbHLH29*	519	58,204.60	6.33	39.60	78.81	−0.447
MSYJ111900.1	*CrbHLH30*	512	56,810.90	5.56	60.06	78.26	−0.538
MSYJ143890.1	*CrbHLH31*	316	35,270.32	7.75	41.30	85.16	−0.345
MSYJ047470.1	*CrbHLH32*	366	40,702.18	8.41	50.28	78.33	−0.541
MSYJ047460.1	*CrbHLH33*	364	40,665.78	6.12	58.04	80.58	−0.477
MSYJ242210.1	*CrbHLH34*	212	23,975.01	9.66	53.17	101.08	−0.320
MSYJ247350.1	*CrbHLH35*	341	37,620.01	7.67	60.58	56.95	−1.002
MSYJ232700.4	*CrbHLH36*	241	26,523.52	5.31	59.72	82.95	−0.619
MSYJ239490.2	*CrbHLH37*	214	24,457.47	5.52	61.03	67.10	−0.946
MSYJ183770.1	*CrbHLH38*	235	25,755.34	6.75	51.52	75.49	−0.514
MSYJ228520.1	*CrbHLH39*	236	25,950.56	8.36	57.32	74.45	−0.553
MSYJ237760.1	*CrbHLH40*	250	27,493.21	5.75	55.86	74.2	−0.506
MSYJ095510.1	*CrbHLH41*	506	56,290.32	7.07	66.52	81.64	−0.418
MSYJ238820.1	*CrbHLH42*	234	26,616.07	6.67	35.94	76.28	−0.711
MSYJ000300.1	*CrbHLH43*	331	37,194.71	4.57	58.16	71.36	−0.802
MSYJ208070.1	*CrbHLH44*	252	28,837.98	7.64	58.62	85.12	−0.437
MSYJ261990.1	*CrbHLH45*	459	51,646.69	6.16	44.83	85.19	−0.434
MSYJ280970.1	*CrbHLH46*	559	61,182.96	5.27	46.09	71.18	−0.606
MSYJ047620.1	*CrbHLH47*	353	39,507.35	5.1	62.24	78.5	−0.490
MSYJ065830.1	*CrbHLH48*	273	31,273.05	4.70	55.38	69.34	−0.638
MSYJ143230.1	*CrbHLH49*	611	68,556.92	4.89	38.49	78.63	−0.543
MSYJ189770.2	*CrbHLH50*	250	28,719.09	8.34	50.89	90.12	−0.36
MSYJ018900.1	*CrbHLH51*	367	40,965.20	5.08	33.48	81.34	−0.374
MSYJ018890.1	*CrbHLH52*	287	31,509.16	5.25	38.47	81.57	−0.497
MSYJ018870.1	*CrbHLH53*	210	23,234.97	4.93	47.42	69.62	−0.655
MSYJ164470.2	*CrbHLH54*	269	29,985.98	5.84	54.59	92.42	−0.339
MSYJ186060.1	*CrbHLH55*	264	28,785.67	6.86	49.62	83.94	−0.423
MSYJ050860.1	*CrbHLH56*	279	31,002.13	8.85	50.67	81.54	−0.468
MSYJ065270.1	*CrbHLH57*	584	65,471.04	8.82	52.46	74.97	−0.530
MSYJ209320.2	*CrbHLH58*	364	40,665.61	6.44	68.19	77.23	−0.711
MSYJ048190.1	*CrbHLH59*	225	25,652.07	9.93	52.35	97.82	−0.371
MSYJ035990.1	*CrbHLH60*	238	26,927.67	7.01	61.37	91.39	−0.405
MSYJ134110.1	*CrbHLH61*	293	31,857.65	6.70	55.86	66.89	−0.472
MSYJ245010.1	*CrbHLH62*	376	40,928.65	4.78	59.06	64.68	−0.769
MSYJ066560.1	*CrbHLH63*	366	40,651.83	5.27	49.31	65.33	−0.706
MSYJ076010.1	*CrbHLH64*	169	19,014.44	7.07	56.25	60.65	−0.615
MSYJ187200.1	*CrbHLH65*	241	26,833.87	5.53	54.51	60.25	−0.663
MSYJ049300.1	*CrbHLH66*	417	46,170.88	5.85	46.31	64.30	−0.746
MSYJ131440.1	*CrbHLH67*	892	102,017.78	6.58	50.21	84.69	−0.306
MSYJ257940.1	*CrbHLH68*	272	29,959.74	8.61	49.68	65.00	−0.518
MSYJ182490.1	*CrbHLH69*	267	29,843.47	9.51	59.70	71.99	−0.456
MSYJ082400.1	*CrbHLH70*	152	17,144.66	10.13	51.79	81.58	−0.513
MSYJ036660.1	*CrbHLH71*	291	32,246.84	6.33	42.26	63.09	−0.686
MSYJ223830.1	*CrbHLH72*	247	27,979.54	5.99	55.07	86.8	−0.533
MSYJ183960.1	*CrbHLH73*	493	53,680.28	6.14	59.63	54.08	−0.640
MSYJ201180.1	*CrbHLH74*	459	499,898.70	6.83	61.56	53.99	−0.688
MSYJ256210.1	*CrbHLH75*	431	46,477.76	6.86	65.44	69.28	−0.468
MSYJ258490.2	*CrbHLH76*	257	27,958.09	5.49	57.57	73.58	−0.585
MSYJ219890.3	*CrbHLH77*	748	82,439.8	5.78	41.78	80.6	−0.366
MSYJ160330.1	*CrbHLH78*	93	10,447.76	7.94	93.56	101.72	−0.490
MSYJ232630.1	*CrbHLH79*	91	10,315.60	9.09	79.13	95.38	−0.652
MSYJ120230.1	*CrbHLH80*	91	10,360.86	9.09	84.39	108.24	−0.413
MSYJ265660.2	*CrbHLH81*	404	45,419.53	6.90	60.68	59.18	−0.910
MSYJ116150.1	*CrbHLH82*	362	39,860.15	9.08	49.78	59.50	−0.744
MSYJ162360.1	*CrbHLH83*	853	93,338.07	7.54	43.95	56.08	−0.807
MSYJ225570.1	*CrbHLH84*	371	39,339.29	8.59	62.21	58.89	−0.599
MSYJ281850.1	*CrbHLH85*	941	103,706.16	8.34	48.03	67.89	−0.493
MSYJ250810.2	*CrbHLH86*	535	58,268.31	6.25	61.16	49.50	−0.717
MSYJ174150.3	*CrbHLH87*	744	79,818.85	5.99	54.57	60.94	−0.638
MSYJ112680.1	*CrbHLH88*	1340	149,772.15	8.88	62.61	63.25	−0.893
MSYJ258060.1	*CrbHLH89*	293	31,542.93	5.16	60.79	69.93	−0.490
MSYJ182580.1	*CrbHLH90*	409	44,239.29	6.33	56.81	75.75	−0.466
MSYJ176470.1	*CrbHLH91*	419	46,235.85	8.62	62.87	54.80	−0.755
MSYJ108410.1	*CrbHLH92*	464	50,717.49	8.51	59.94	55.75	−0.638
MSYJ074340.1	*CrbHLH93*	461	48,308.26	5.61	47.01	62.08	−0.590
MSYJ243680.2	*CrbHLH94*	338	35,982.21	5.43	62.20	69.67	−0.451
MSYJ219480.1	*CrbHLH95*	294	31,303.42	5.85	55.82	79.35	−0.352
MSYJ122980.1	*CrbHLH96*	519	56,318.99	4.96	51.52	80.42	−0.462
MSYJ277260.1	*CrbHLH97*	396	44,630.71	6.22	56.88	55.93	−0.784
MSYJ277250.1	*CrbHLH98*	279	31,322.64	8.90	50.40	73.15	−0.313
MSYJ265030.2	*CrbHLH99*	341	38,175.63	6.35	57.82	67.24	−0.862
MSYJ038780.1	*CrbHLH100*	340	37,950.64	5.45	64.05	55.38	−0.945
MSYJ177500.1	*CrbHLH101*	565	61,509.43	9.01	51.53	58.73	−0.829
MSYJ280900.2	*CrbHLH102*	370	41,598.09	6.14	42.93	60.68	−0.801
MSYJ066230.1	*CrbHLH103*	480	52,613.39	7.12	45.28	67.02	−0.602
MSYJ116110.1	*CrbHLH104*	756	83,682.97	5.99	45.16	74.30	−0.590
MSYJ090140.1	*CrbHLH105*	553	60,065.44	6.06	51.20	61.81	−0.724
MSYJ161620.1	*CrbHLH106*	277	31,230.58	8.86	40.73	78.92	−0.495
MSYJ232170.1	*CrbHLH107*	276	29,821.98	5.51	53.51	60.87	−0.697
MSYJ287560.1	*CrbHLH108*	256	28,558.69	6.37	55.97	55.62	−0.927
MSYJ221570.1	*CrbHLH109*	72	8430.11	9.46	39.93	124.17	0.266
MSYJ208710.3	*CrbHLH110*	558	59,511.88	5.90	53.72	58.41	−0.672
MSYJ221550.1	*CrbHLH111*	481	52,707.83	7.03	49.35	65.68	−0.696
MSYJ174650.1	*CrbHLH112*	433	48,390.10	6.29	54.59	65.57	−0.718
MSYJ037560.2	*CrbHLH113*	363	41,522.10	8.91	48.23	63.91	−0.567
MSYJ133760.1	*CrbHLH114*	242	26,942.30	6.39	48.64	65.29	−0.510
